# Catechin-Albumin Conjugates: Enhanced Antioxidant Capacity and Anticancer Effects

**DOI:** 10.1155/2022/1596687

**Published:** 2022-10-08

**Authors:** Tooru Ooya, Izumi Haraguchi

**Affiliations:** ^1^Department of Chemical Science and Engineering, Graduate School of Engineering, Kobe University, Kobe, Japan; ^2^Center for Advanced Medical Engineering Research & Development (CAMED), Kobe University, Kobe, Japan

## Abstract

(+)-Catechin conjugated with human serum albumin (CT–HSA) was prepared and evaluated as a drug carrier bearing anticancer effects. It was found that 2.4 mol of CT was conjugate to 1 mol HSA. The CT–HSA has an antioxidant capacity of about 3.3 times the amount of CT in the conjugate. Intracellular incorporation of the CT–HSA was analyzed by fluorescence-activated cell sorting (FACS) and confocal laser scanning microscopy (CLSM) measurements using fluorescein isothiocyanate (FITC)-labelled CT–HSA. The results indicated that the FITC-labelled CT–HSA was incorporated into HeLa cells in a concentration-dependent manner. The CT–HSA enhanced the binding of anticancer drugs (5-fluorouracil (5-Fu) and mitomycin C (MMC)) comparing with HSA, and the CT–HSA mixed with 5-Fu or MMC decreased significantly the HeLa cell viability as compared with the same concentration of each drug. In addition, intracellular reactive oxygen species (ROS) scavenging by the CT–HSA is likely to affect the anticancer effects. Thus, the CT–HSA enhanced anticancer drug efficacy in relation to controlling ROS-scavenging ability.

## 1. Introduction

**Figure 1:**
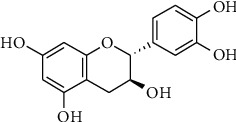
Structural image of (+)-catechin (CT).

(+)-Catechin (CT; Figure 1) is not only an antioxidant chemical that bears radical scavenging ability derived from its phenol moieties but also a cellular DNA breakage agent that acts by intercalation. For example, (+)-catechin-loaded ninosome has been proposed as a potential carrier for transdermal delivery of antioxidants [1]. Furthermore, in the last decade, the coadministration of anticancer drugs with various antioxidants improved their therapeutic effects in humans. For example, in the case of doxorubicin (DOX), in 2004, Mei et al. reported that catechins are effective in suppressing the extracellular diffusion of DOX by inhibiting the activity of the drug-resistant P-glycoprotein [2]. Recently, tea polyphenol was subjected to coat calcium phosphate-based nanospheres for DOX deliver [3], which is based on good biodegradability of the polyphenol in response to the high glutathione in cancer cells [4, 5]. It has also been reported that catechins enhance this effect by regulating the intracellular reactive oxygen species (ROS) induced by DOX [6]. Furthermore, Chen et al. have reported that the administration of epigallocatechin gallate and sulforaphane in combination with paclitaxel-resistant human breast cancer cells does not induce the expression of the telomerase subunits hTERT and Bcl-2 and induces apoptosis [7]. However, cellular uptake and intracellular trafficking have not been controlled to date because of the lack of drug carriers.

Human serum albumin (HSA) is one of the important proteins in the serum. HSA has drug-binding sites and has been used as a carrier of various drugs [8–10] and as model protein drug [11]. Moreover, according to a review compiled by Elzoghby et al. in 2012, HSA can pass through the vascular endothelium because of the gp60 receptor, which binds to HSA in vascular endothelial cells [12]. In addition, HSA is selected for cancer cells because the extracellular matrix that surrounds them contains more secreted protein acidic and rich in cysteine/osteonectin, which binds to HSA, than do normal cells. HSA has been put into practical use as a drug carrier for poorly water-soluble anticancer drugs. For example, Abraxane®, which is a mixture of HSA and paclitaxel, was used for lung cancer therapy [13, 14]. Approval for pancreatic cancer has been obtained, and further expansion of indications is expected in the future. Thus, albumin-based drug carriers have been extensively studied to date [15], and albumin can bind anticancer drugs [16], imaging probes [17, 18], nitric oxide [19], porphyrin [20], etc. However, HSA as a drug carrier has still some disadvantages such as low efficacy of drugs due to low accumulation [21] and unwanted diffusion drugs that induces side effects in normal cells [22]. Chemical modification of HSA has been considered to improve the disadvantage of HSA. For example, conjugation with mannose provides vascular targetability of HSA [23]. Arg-Gly-Asp (RGD) peptide modification to HSA provides transcellular transport ability [24, 25]. We hypothesize that the modification of albumin by catechins may lead to the attribution of their own biological functions to the albumin-based drug carriers. In this research, a CT–HSA conjugate was prepared to evaluate the synergistic effect of catechin accumulation and HSA-based anticancer drugs, 5-fluorouracil (5-Fu), and mitomycin C (MMC), to a cultured cancer cell.

## 2. Experimental

### 2.1. Materials

HSA, l-(+)-ascorbic acid, mitomycin C (MMC), and ethanol were purchased from FujiFilm Wako Chemical Industries, Ltd. (Osaka, Japan). d-(+)-Catechin (CT), 5-fluorouracil (5-Fu), Folin-Ciocalteu reagent (FCR) solution, Dulbecco's modified Eagle's medium (DMEM), Dulbecco's phosphate-buffered saline (DPBS), 0.25% trypsin/1 mM EDTA solution, penicillin-streptomycin mixed solution, sodium carbonate, and fluorescein isothiocyanate isomer I (FITC) were purchased from Nacalai Tesque, Inc. (Kyoto, Japan). Hydrogen peroxide was purchased from Kanto Chemical Co., Inc. (Tokyo, Japan). 1,1-Diphenyl-2-picrylhydrazyl free radical (DPPH) was purchased from Tokyo Chemical Industry Co., Ltd. (Tokyo, Japan). Fetal bovine serum (FBS) was purchased from Sigma-Aldrich Co. LLC. (St. Louis, U.S.A). The 2′,7′-dichlorodihydrofluorescein diacetate (DCFH-DA) was purchased from Funakoshi Co., Ltd. (Tokyo, Japan).

### 2.2. Preparation of the CT–HSA Conjugate

**Figure 2:**
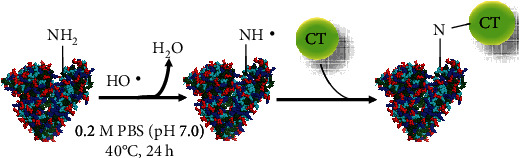
Schematic depiction of human serum albumin (HSA) conjugated with catechin (CT).

The CT–HSA conjugate was prepared by a free radical method, with some alterations [26]; HSA (0.1 g), CT (0.05 g), H_2_O_2_ (18 *μ*L), and ascorbic acid (0.03 g) were dissolved in 0.2 M PBS (pH 7.4) (30 mL) and stirred under an N_2_ atmosphere at 40°C for 24 h (Figure 2). The resulting solution was dialyzed (MWCO = 12, 000 − 14,000) against distilled water at 25°C for 24 h. During this incubation, the medium was exchanged twice. After the solution was concentrated by distilling off under reduced pressure, unreacted CT and any other compounds were separated by gel permeation chromatography (GPC) at room temperature under gravity flow of water on Glass Econo-Column® Columns (*Φ*1.0 × 30 cm; Bio-Rad Laboratories, CA, USA) packed with Sephadex® G-75. The recovered fractions were collected and lyophilized for 24 h.

### 2.3. Quantification of CT Amount by the Folin-Ciocalteu Reagent (FCR) Method and MALDI-TOF Mass Spectroscopy

The CT amount in the CT–HSA conjugate was quantified, followed by the application of the FCR method [27]. In order to calculate the CT amount in the conjugate, a calibration curve of free CT was used. The calibration curve was made as follows: CT (0.71 mg, 2.3 × 10^−6^ mol) was dissolved in 19 mL of distilled water to prepare 120 *μ*M CT stock solution. By the dilution of this CT stock solution, CT solutions of various concentrations (8, 16, 24. 32, 40, 60, and 80 *μ*M) were prepared. To 0.6 mL of each concentration of CT solution, FCR reagent (0.10 mL) was added, vortexed, and allowed to stand for 3 minutes. Then, 2.0 % sodium carbonate solution (1.5 mL) was added, vortexed, and allowed to stand for 2 hours. The absorbance at 750 nm of each solution was recorded by the VIS measurements, giving the calibration curve. From the obtained calibration curve, we calculated slope, the correlation coefficient (*R*^2^ = 0.995), and intercept of the regression equation via the least squares method (Equation (1)):

(1) $$\begin{array}{c} \text{CT equivalent}\left(\mu \text{M}\right)=2.64\times 102\times \left(\text{Abs at wave length }750\text{nm}\left(-\right)\right)–0.37. \end{array}$$

The CT–HSA conjugate (1 mg) was dispersed in pure water (0.6 mL). After dissolution, 0.1 mL of FCR was added to the solution and mixed thoroughly. After 3 min of incubation, 0.3 mL of 2% Na_2_CO_3_ were dropped, and the solution was kept for 2 h. Then, the absorbance at 750 nm was recorded by the VIS measurements. The HSA solution in the same concentration was used as a control. The difference in absorbance between the CT–HSA conjugate and HSA (Δ*A*) is considered the increase in phenolic -OH groups of the conjugate due to the binding of CT to HSA. Therefore, the increase in phenolic -OH groups of the conjugate was calculated in terms of the amount of CT substance by applying the following equation (Equation (2));

(2) $$\begin{array}{c} \text{CT equivalent}\left(mol/mol\;dry\;HSA\right)=\left(2.64\times 10^{2}\times ∆A-0.37\right)\times \left(0.6\times 10^{-3}\right)\times M_{n}\times 10^{-3}, \end{array}$$

where 0.6 × 10^−3^ is the volume of solution (L), *M*_*n*_ is molecular weight of HSA (66,000), and 10^−3^ is mass of the conjugate used (g).

MALDI-TOF mass spectra of the CT–HSA conjugate and the native HSA were measured using a MALDI-TOF MS apparatus (Voyager-DE™-1000, AB SCIEX, Tokyo, Japan). As a matrix, a sinapinic acid aqueous solution containing 50% acetonitrile was used, pH of which was adjusted to be 2 using trifluoroacetic acid.

### 2.4. Evaluation of the Antioxidant Properties of the Conjugate

The antioxidant capacities of the CT–HSA were assessed based on its DPPH radical scavenging ability. The DPPH assay was conducted according to a previous protocol [28] with some modifications. This test was performed on the CT–HSA conjugate using HSA as a control. The CT–HSA conjugate (0.6 mg) was dispersed in 2 mL of 0.2 M acetic acid buffer (pH 5.0), followed by the addition of 2 mL of a DPPH solution dissolved in ethanol (1.4 × 10^−4^ M). The HSA (0.6 mg) solution was prepared in the same conditions. Each tube was allowed to stand in the dark at RT. Thirty minutes later, the residual DPPH concentration was determined by the VIS measurements at 525 nm. The CT equivalent antioxidant of the CT–HSA conjugate was calculated by using a calibration curve of free CT (5, 10, 15, 20, 25, and 30 *μ*M). Inhibition (%), which is the decrease (%) in DPPH absorbance, was calculated by the following equation:

(3) $$\begin{array}{c} \text{inhibition }\left(\%\right)=\frac{A_{0}-A_{1}}{A_{0}}\times 100, \end{array}$$

where *A*_0_ is the absorbance of a standard prepared in the same conditions without adding any polymers and *A*_1_ is the absorbance of the sample solution.

### 2.5. Binding Analysis of Anticancer Drugs to the CT–HSA Conjugate

The binding of 5-fluorouracil (5-Fu) and mitomycin C (MMC) to the conjugate was evaluated by measurements of the fluorescent quenching attributed to tryptophan in HSA [29, 30]. In this method, hydrophobic agents could interact with the hydrophobic cavity of subdomain IIA in proximity to the Trp214 residue, causing fluorescence quenching of Trp214. We performed fluorescence measurements in a path length quartz cuvette (1 × 1 cm) at 25°C. The fluorescence emission spectra (e.g., 280 nm) were collected in the wavelength range of 300-500 nm. The concentration of both HSA and CT–HSA was 3.0 *μ*M, which were used together with 5-Fu (0-400 *μ*M) or MMC (0-14 *μ*M) in the measurements. All samples were prepared by dissolution in 10 mM PBS.

### 2.6. Preparation of a CT–HSA Conjugate Labelled with FITC

To observe the intracellular incorporation of the CT–HSA conjugate, we prepared a CT–HSA conjugate labelled with FITC. FITC-labelled HSA was prepared as follows; HSA (50 mg) and FITC (5 mg) dissolved in 5 mL of 0.1 M carbonate-bicarbonate buffer (pH 9.0) were allowed to stand at 4°C for 8 h. Then, unreacted FITC was removed by GPC, similar to that described for the CT–HSA conjugate preparation. The collected fraction was frozen and dried with a freeze drier for 24 h, to obtain the FITC-labelled HSA. To prepare the FITC-labelled CT–HSA, the resultant product (50 mg), CT (46 mg), H_2_O_2_ (17 *μ*L), and ascorbic acid (23 mg) were dissolved in 30 mL of 0.2 M PBS (pH 7.4) and stirred under an N_2_ atmosphere at 40°C for 24 h. Finally, unreacted CT and any other compounds were separated by GPC. The resulting product solutions were lyophilized for 24 h.

### 2.7. In Vitro Assay

#### 2.7.1. Cell Culture

HeLa cell (TKG0331, Deposited from Tohoku Univ., Japan) culture was performed using DMEM containing 10% FBS and 1% penicillin-streptomycin as a cell culture medium. The CO_2_ incubator was set at 37°C with 5% CO_2_.

#### 2.7.2. Intracellular Uptake of the CT–HSA Conjugate

HeLa cells were seeded into a 6-well plate at 1.0 × 10^4^ cells/well (2 mL) and were allowed to adhere for 24 h. On the following day, the cells were treated with the FITC-labelled CT–HSA conjugate or FITC-labelled HSA (1-100 *μ*M, 100 *μ*L) dissolved in DPBS. For 24 h of incubation, the cells were washed with DPBS and resuspended in 0.5 mL of DPBS. Intracellular fluorescence corresponding to the incorporated FITC-labelled CT–HSA conjugate was detected by FACS (BD FACSCANTO II, BD Biosciences, U. S. A.) (Used GFP filter; Ex: 488 nm, Em: 530/30 nm). Moreover, intracellular incorporation was observed by confocal laser scanning microscopy (CLSM: Fluo View™ FV1000, Olympus, Japan). In this case, HeLa cells were seeded into a 35 mm glass dish at 2.0 × 10^4^ cells/well (2 mL) and were allowed to stand for 24 h. On the following day, the cells were treated with the FITC-labelled CT–HSA conjugate (100 *μ*M, 100 *μ*L) or FITC-labelled HSA (100 *μ*M, 100 *μ*L) dissolved in DPBS. For 24 h of incubation, the cells were washed, and the cell culture medium was replaced with DPBS. Subsequently, CLSM measurements were carried out: FITC was excited at 488 nm (argon laser) and emission was passed through a bandpass filter (500-550 nm) before imaging. The measurement conditions were as follows: PMT voltage: 700 V, pinhole size: 145 *μ*m, and sampling speed: ,2.0 *μ*s/pixel.

#### 2.7.3. Cytotoxicity Assay

HeLa cells were seeded into a 96-well plate at 5.0 × 10^3^ cells/well (100 *μ*L) and were allowed to adhere at 37°C for 24 h. On the following day, the cells were treated with the CT–HSA conjugate or HSA (5-100 *μ*M, 10 *μ*L) mixed in DPBS with each of the following anticancer drugs: MMC (2 *μ*M) or 5-Fu (10 *μ*M). For 24 h of incubation, CCK-8 (10 *μ*L) was mixed in each well. After another 2 h incubation, the absorbance 450 nm was recorded by using a microplate reader (Corona Grating Microplate Reader SH-9000 Series, Corona Electric Co., Ltd, Japan). The cell viability was calculated by the following equation:

(4) $$\begin{array}{c} \text{Cell viability }\left(\%\right)=\frac{\left(A_{s}-A_{s_{0}}\right)}{\left(A_{c}-A_{c_{0}}\right)}\times 100, \end{array}$$

where *A*_*s*_ is the absorbance of the well solution in the presence of both the sample and cells and *A*_*s*_0__ is the absorbance of the well solution in the presence of the sample without HeLa cells.

### 2.8. Intracellular ROS Measurements

The amount of intracellular ROS species was conducted by fluorescence measurements based on the oxidative convention of DCFH-DA to 2′,7′-dichlorofluorescein (DCF) [31]. HeLa cells were seeded into a 96-well black plate at 1.0 × 10^4^ cells/well (100 *μ*L) and were allowed to adhere for 24 h. On the following day, the culture medium was substituted with 10 *μ*M DCFH-DA solution (100 *μ*L) dissolved in DMEM. One hour later, the culture medium was substituted with of the CT–HSA conjugate or HSA (100 *μ*M) mixed in DMEM with each of the following anticancer drugs: MMC (2 *μ*M) or 5-Fu (10 *μ*M). After 24 h incubation, fluorescence was measured using a microplate reader (Corona Grating Microplate Reader SH-9000 Series, Corona Electric Co., Ltd, Japan) with 485 nm excitation and 538 nm emission. The ROS production (%) was calculated by the following equation:

(5) $$\begin{array}{c} \text{ROS production }\left(\%\right)=\left(F_{s}-F_{s_{0}}\right)/F_{c}\times 100, \end{array}$$

where *F*_*s*_ is the fluorescence of the solution in the presence of both the compounds and cells, *F*_*s*_0__ is the fluorescence of the solution in the presence of the compounds without HeLa cells, and *F*_*c*_ is the fluorescence of the solution in the presence of HeLa cells without compounds.

### 2.9. Statistical Analysis

Statistical analysis was performed by Student's *t*-test as a post hoc comparison. Significance level was set at *p* < 0.05 or *p* < 0.01.

## 3. Results and Discussion

### 3.1. Preparation of the CT–HSA Conjugate

**Figure 3:**
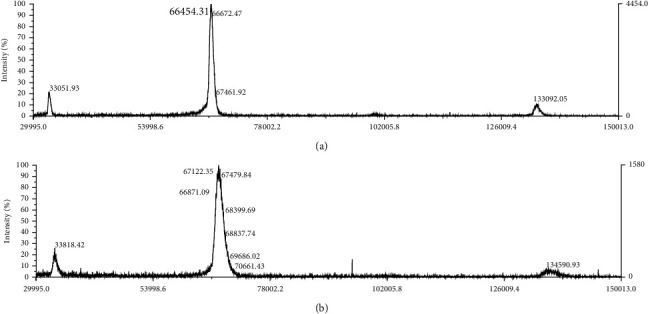
MALDI-TOF mass spectra of (a) native HSA and (b) the CT–HSA conjugate.

The CT introduction to HSA molecule could be evaluated by the reducing ability of CT in comparison with the CT–HSA conjugate to form (PMoW_11_O_40_)^4-^ that was a quantitatively measurable compound on the FCA method. Consequently, we determined that 1.0 mol of the CT–HSA conjugate contains 2.33 mol of CT. This amount was well correlated with the results of the MALDI-TOF mass measurements. A peak attributed to native HSA was observed at *m*/*z* = 66,454 (M+Na) (Figure 3(a)). After the conjugation, the peak at *m*/*z* = 67,122 (M+Na) was observed (Figure 3(b)). From this result, we calculated that the CT–HSA conjugate contains 2.37 mol of CT.

To assess the antioxidant activity of CT in the CT–HSA conjugate, the DPPH radical was assessed [32]. The antioxidant activity of the CT–HSA conjugate was evaluated in terms of DPPH reduction using CT as a reference compound, and data are expressed as inhibition. The antioxidant activity of 1.0 mol of the CT–HSA conjugate was calculated to be 7.82 mol of CT equivalent.

According to the results obtained via both Folin-Ciocalteu assay and DDPH assay, the CT–HSA conjugate has an antioxidant capacity of about 3.3 times the amount of CT in the conjugate. It has been reported that antioxidant capacity increases when antioxidants are combined with macromolecules [33, 34]. Ihara et al. reported that, when catechin was conjugated to poly(lysine), the inhibitory rate of collagen and hyaluronic acid degradation and the inhibition rate of ROS-generating xanthine oxidase activity were significantly improved compared with catechin alone [34]. In addition, when catechin was bound to ovotransferrin, the oxygen radical absorption capacity was about twice that of catechin alone [33]. Although the mechanism underlying this phenomenon has not been clarified, the localized concentration of polyphenols causes coupling with nearby phenols, which enhances antioxidant capacity [35].

### 3.2. Binding Interaction of Anticancer Drugs to the CT–HSA Conjugate

We evaluated the binding interaction of anticancer drugs to CT–HSA based on the binding constant (*K*_*b*_) (Table 1). The quantitative evaluation of *K*_*b*_ was performed using an analysis of the fluorescence quenching data (see: Supporting Information (SI) Figures S1–S4) based on the following equation (Stern-Volmer plot: see: Supporting Information (SI) Figure S5).

(6) $$\begin{array}{c} \log\frac{F_{0}-F}{F}=\log K_{b}+\text{n }\log\left[Q\right], \end{array}$$

where *F*_0_ and *F* are the steady-state fluorescence intensities of HSA in the absence/presence of each anticancer drug, respectively, *n* is a set of equivalent sites, and [*Q*] is the concentration of each anticancer drug.

The data listed in Table 1 suggest that the reliability of the results was high because the binding constants and the number of binding sites of HSA to each anticancer drug were in the same order as the literature values [29, 30]. Based on this result, it was shown that CT–HSA has a larger binding constant to each anticancer drug compared with has alone. This might be attributed to the fact that the affinity between CT and each anticancer drug existing in or near the drug-binding site of HSA was improved by *π* − *π* stacking. It was also shown that CT–HSA has more binding sites to each anticancer drug than does has, which suggests that the drug site of each conjugate might have promoted intermolecular interaction via the introduction of CT. The results reported above showed that CT–HSA has ability as a drug carrier without losing the drug-binding ability, even after the introduction of CT.

### 3.3. Intracellular Incorporation of the CT–HSA Conjugate

**Figure 4:**
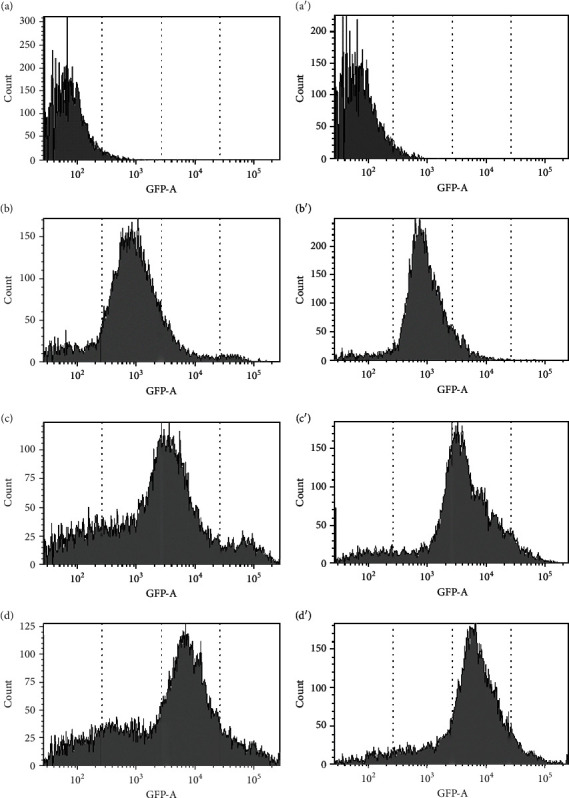
Intracellular incorporation of FITC-labelled HSA ((a) original fluorescence of HeLa cells, (b) 5 *μ*M, (c) 50 *μ*M, and (d) 100 *μ*M) or FITC-labelled CT–HSA ((a′) original fluorescence of HeLa cells, (b′) 5 *μ*M, (c′) 50 *μ*M, and (d′) 100 *μ*M). The data were obtained using a flow cytometric analysis.

**Figure 5:**
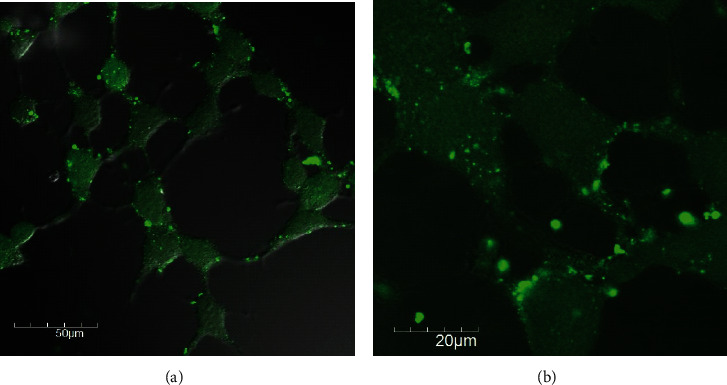
Intracellular incorporation of (a) FITC-labelled HSA (100 *μ*M) or (b) FITC-labelled CT–HSA (100 *μ*M). This observation was performed using confocal laser scanning microscopy.

To analyze the intracellular incorporation of the CT–HSA conjugate compared with has one, FCAS measurements were carried out using FITC-labelled CT–HSA and FITC-labelled HSA in the concentration range of 5-100 *μ*M (Figure 4). As show in Figure 4, both the FITC-labelled CT–HSA and FITC-labelled HSA were incorporated into HeLa cells in a concentration-dependent manner, suggesting the albumin-oriented cellular uptake to HeLa cells [36]. We also conducted the FACS measurements 3-24 h after adding 10 *μ*M of FITC-labelled CT–HSA and FITC-labelled HSA to HeLa cells (Supporting Information (SI) Figures S6). The mean fluorescence of FITC-labelled CT–HSA increased from 2040 at 3 h to 4310 at 24 h, while that of FITC-labelled HSA increased from 2029 at 3 h to 3531 at 24 h. These results suggest that the cellular uptake was governed by HSA-based uptake, and CT was not likely to modulate the cellular uptake. In addition, Figure 5 shows intracellular incorporation via CLSM. These results also supported the results of the FACS measurements: that FITC-labelled CT–HSA and FITC-labelled HSA were taken up into cells in the same manner as HSA.

### 3.4. Evaluation of the Anticancer Activity of 5-Fu and MMC Mixed with CT–HSA

**Figure 6:**
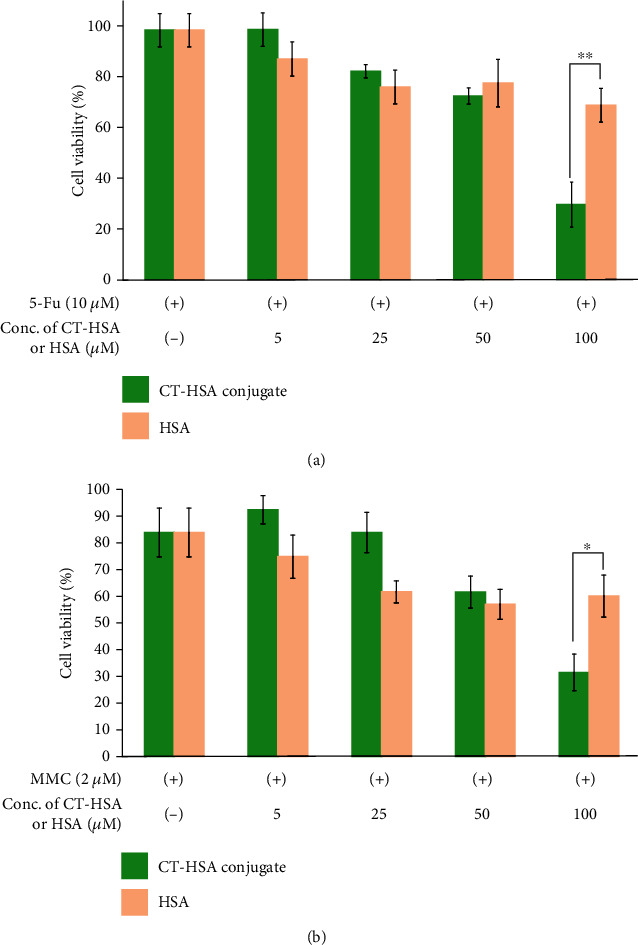
Cytotoxic effect of the CT–HSA conjugate or HSA as a drug carrier on HeLa cells. HeLa cells were treated with the CT–HSA conjugate (0-100 *μ*M) or HSA (0-100 *μ*M) mixed with (a) 10 *μ*M 5-Fu and (b) 2 *μ*M MMC. Differences between each concentration of the sample and the control were analyzed by *t*-test (mean ± SD, *n* = 5); ^∗^*p* < 0.01.

**Figure 7:**
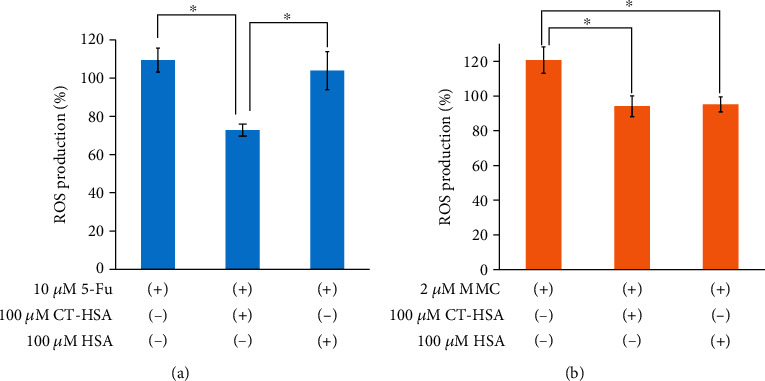
Intracellular ROS inhibition in HeLa cells treated with the CT–HSA conjugate (100 *μ*M) or HSA (100 *μ*M) together with (a) 5-Fu (10 *μ*M) or (b) MMC (2 *μ*M). The differences between each sample were analyzed by *t*-test (mean ± SD, *n* = 5); ^∗^*p* < 0.05.

The effect of the CT–HSA conjugate combined with 5-Fu or MMC on cell viability was evaluated (Figure 6). Here, in the case of HSA, when its concentration was increased to 100 *μ*M, the cell viability was suppressed to about 70%. Conversely, in the case of the CT–HSA system, the survival rate of HeLa cells was suppressed to 30% for 5-Fu and to 37% by MMC at a concentration of 100 *μ*M. Because the CT-has conjugate itself had no cytotoxicity (see: Supporting Information (SI) Figure. S7), the decreased cytotoxicity observed for the drug combinations was attributed to the 5-Fu and MMC bound to CT–HSA. Regarding the higher cytotoxicity of the anticancer drugs in the presence of 100 *μ*M CT–HSA, we investigated the possibility that the conjugate scavenges reactive oxidant species. We hypothesized that ROS-scavenging activity is possibly correlated with the anticancer drug activity. In general, it is known that intracellular ROS are generated when an anticancer drug is added to cells [37]; however, it has been reported that these ROS suppress the effect of the anticancer drug [6]. Therefore, it was thought that, after the combination of CT–HSA with an anticancer drug, the effect of the anticancer drug is enhanced by eliminating ROS because of the antioxidant capacity of CT.  Figure 7(a) shows the intracellular ROS incidence rate when 5-Fu and each sample were mixed and added to HeLa cells. The mixed system of CT–HSA and 5-Fu significantly suppressed the ROS incidence rate by 36% compared with 5-Fu alone. A significant difference (*p* < 0.05) was also confirmed between the mixed system of HSA and 5-Fu and CT–HSA. Together with the result presented in Figure 6, this showed that, when 5-Fu and CT–HSA are mixed and added to HeLa cells, the intracellular ROS generated from 5-Fu are eliminated by the antioxidant capacity of CT–HSA. Furthermore, it was suggested that the cell viability was suppressed as a result of the increase in the effect of 5-Fu. Conversely, Figure 7(b) shows the intracellular ROS incidence rate when MMC and each sample were mixed and added to HeLa cells. When MMC and CT–HSA were mixed, the incidence of intracellular ROS was significantly suppressed by about 25% compared with MMC alone. As no significant difference was found between CT–HSA and HSA, we considered that the involvement of the CT–HSA-derived ROS-scavenging ability and the suppression of cell viability were low in the system containing MMC. These results suggest that 5-Fu and MMC induce cancer cell death via different mechanisms.

It is known that 5-Fu is an anticancer drug that acts specifically in the S phase of the cell cycle [38]. Furthermore, ROS generation in cells inhibits cell cycle progression during S phase [39]. As shown in Figure 6(b), the CT–HSA conjugate mixed with 5-Fu decreased the ROS production. From this result, we can imagine that DNA synthesis is restored by the CT–HSA conjugate mixed with 5-Fu. In the recovered DNA synthesis condition, 5-Fu can act as an inhibitor of DNA synthesis, presumably due to the increased the number of cells in the S phase. Thus, 5-Fu was considered to have higher cytotoxicity. Conversely, MMC exerts its cytotoxicity by intercalating with DNA, suggesting that no correlation with ROS elimination is present. As MMC had a high binding constant with CT–HSA, it is considered that a large amount of MMC was taken up into cells after the formation of the complex. Subsequently, after the degradation of CT–HSA by intracellular lysosomes, the released MMC intercalates into DNA; however, CT also intercalates DNA [40], which is considered to have increased cytotoxicity.

## 4. Conclusion

In this study, the (+)-catechin was conjugated with human serum albumin (CT–HSA), and the HSA modification was found to enhance the binding of anticancer drugs, 5-fluorouracil (5-Fu) and mitomycin C (MMC). CT–HSA has an antioxidant capacity of about 3.3 times the amount of CT in the conjugate. The CT–HSA and the drug mixture significantly decreased the cell viability of HeLa cells as compared with the same concentration of drug. The CT–HSA had the properties of intracellular ROS scavenging, leading to enhancing the anticancer effects of 5-Fu in conjunction with cell cycle-related inhibition of DNA synthesis. The CT–HSA also binds with MMC, resulting in the enhanced cellular uptake and interaction with DNA. Therefore, the CT–HSA is expected as a good candidate of drug carrier bearing anticancer effects.

## Figures and Tables

**Table 1:** Binding constant (*K*_*b*_) and number of binding sites (*n*) between 5-Fu or MMC and HSA, CT–HSA.

	*K*_*b*_ (×10^3^ M^−1^)		*n*	
	5-Fu	MMC	5-Fu	MMC
HSA ^[Ref.data]^	0.759 [30]	27.1 [29]	0.74 [30]	1.02 [29]
HSA	0.418	59.4	0.91	1.00
CT–HSA	14.8	192.9	1.35	1.11

## Data Availability

The data are available upon request.

## Supplementary Materials

Supplementary Materials Figure S1: fluorescence emission spectra of HSA in DPBS with the presence of 5-Fu. Figure S2: fluorescence emission spectra of CT–HSA conjugate in DPBS with the presence of 5-Fu. Figure S3: fluorescence emission spectra of HSA in DPBS with the presence of MMC. Figure S4: fluorescence emission spectra of CT–HSA in DPBS with the presence of MMC. Figure S5: Stern-Volmer plots of the binding constant of 5-Fu with HSA, 5-Fu with CT–HSA, MMC with HSA, and MMC with CT-HSA. Figure S6: time dependent of intracellular incorporation of FITC-labelled HSA to HeLa cells (A; 3 h, B; 7 h, and C; 24 h) or FITC-labelled CT–HSA to HeLa cells (A'; 3 h, B'; 7 h, and C'; 24 h). Figure S7: cytotoxic effect of CT and CT–HSA on HeLa cells.
