# Supplementary material for: Catechin-Albumin Conjugates: Enhanced Antioxidant Capacity and Anticancer Effects
Source: Int J Food Sci. 2022 Oct 8;2022:1596687. doi: 10.1155/2022/1596687 (PMC9569216; doi:10.1155/2022/1596687)
Supplement: Supplementary Materials — Figure S1: fluorescence emission spectra of HSA in DPBS with the presence of 5-Fu. Figure S2: fluorescence emission spectra of CT–HSA conjugate in DPBS with the presence of 5-Fu. Figure S3: fluorescence emission spectra of HSA in DPBS with the presence of MMC. Figure S4: fluorescence emission spectra of CT–HSA in DPBS with the presence of MMC. Figure S5: Stern-Volmer plots of the binding constant of 5-Fu with HSA, 5-Fu with CT–HSA, MMC with HSA, and MMC with CT-HSA. Figure S6: time dependent of intracellular incorporation of FITC-labelled HSA to HeLa cells (A; 3 h, B; 7 h, and C; 24 h) or FITC-labelled CT–HSA to HeLa cells (A'; 3 h, B'; 7 h, and C'; 24 h). Figure S7: cytotoxic effect of CT and CT–HSA on HeLa cells. [file 1596687.f1.docx]

***Supplementary Materials***

**Catechin-Albumin Conjugates: Enhanced Antioxidant Capacity and Anticancer Effects**

Tooru Ooya^a,b^* and Izumi Haraguchi^a^

*^a^Department of Chemical Science and Engineering, Graduate School of Engineering, Kobe University, Kobe, Japan*

*^b^ Center for Advanced Medical Engineering Research & Development (CAMED), Kobe University, Kobe, Japan*

*To whom correspondence should be addressed;

E-mail: ooya@tiger.kobe-u.ac.jp
Tel.: +81-78-803-6255; Fax: +81-78-803-6255.


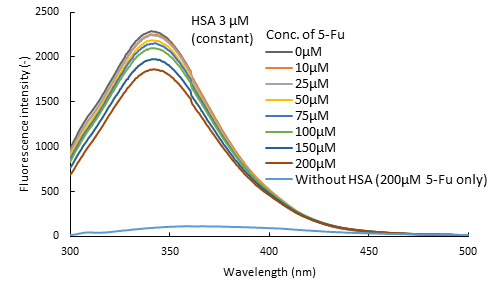


**Figure S1.** Fluorescence emission spectra of HSA (3.0 μM) in DPBS with the presence of 5-Fu at concentration of 0~200 μM. From upper to lower, concentration of 5-Fu was higher. The lowest spectrum was the 5-Fu (200 μM) original fluorescence without HSA. The spectra were recorded at 298 K under excitation of 280 nm

**Figure S2.** Fluorescence emission spectra of CT-HSA conjugate (3.0 μM) in DPBS with the presence of 5-Fu at concentration of 0~200 μM. From upper to lower, concentration of 5-Fu was higher. The lowest spectrum was the 5-Fu (200 μM) original fluorescence without CT-HSA. The spectra were recorded at 298 K under excitation of 280 nm


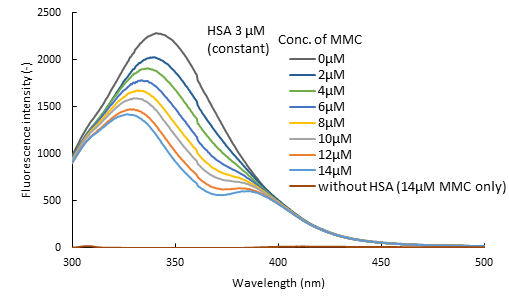


**Figure S3.** Fluorescence emission spectra of HSA (3.0 μM) in DPBS with the presence of MMC at concentration of 0~14 μM. From upper to lower, concentration of MMC was higher. The lowest spectrum was the MMC (14 μM) original fluorescence without HSA. The spectra were recorded at 298 K under excitation of 280 nm.

**Figure S4.** Fluorescence emission spectra of CT-HSA (3.0 μM) in DPBS with the presence of MMC at concentration of 0~14 μM. From upper to lower, concentration of MMC was higher. The lowest spectrum was the MMC (14 μM) original fluorescence without CT-HSA. The spectra were recorded at 298 K under excitation of 280 nm.

(a) (b)


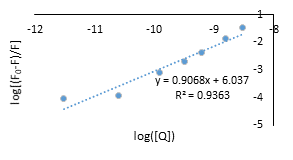

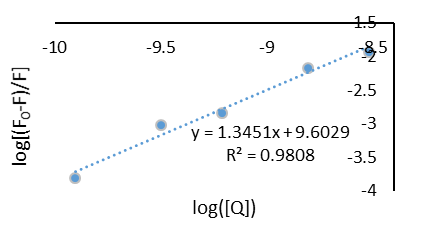


(c) (d)


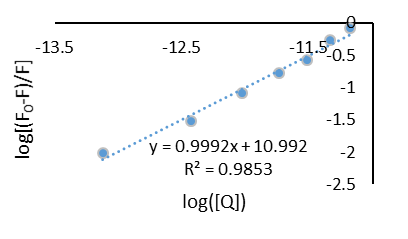

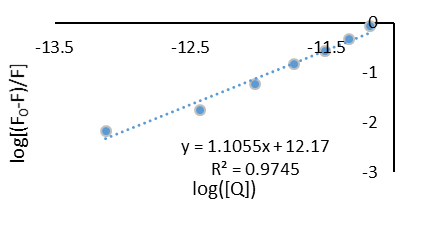


**Figure S5.** Stern-Volmer plots of the binding constant of (a) 5-Fu with HSA, (b) 5-Fu with CT-HSA, (c) MMC with HSA, and (d) MMC with CT-HSA.


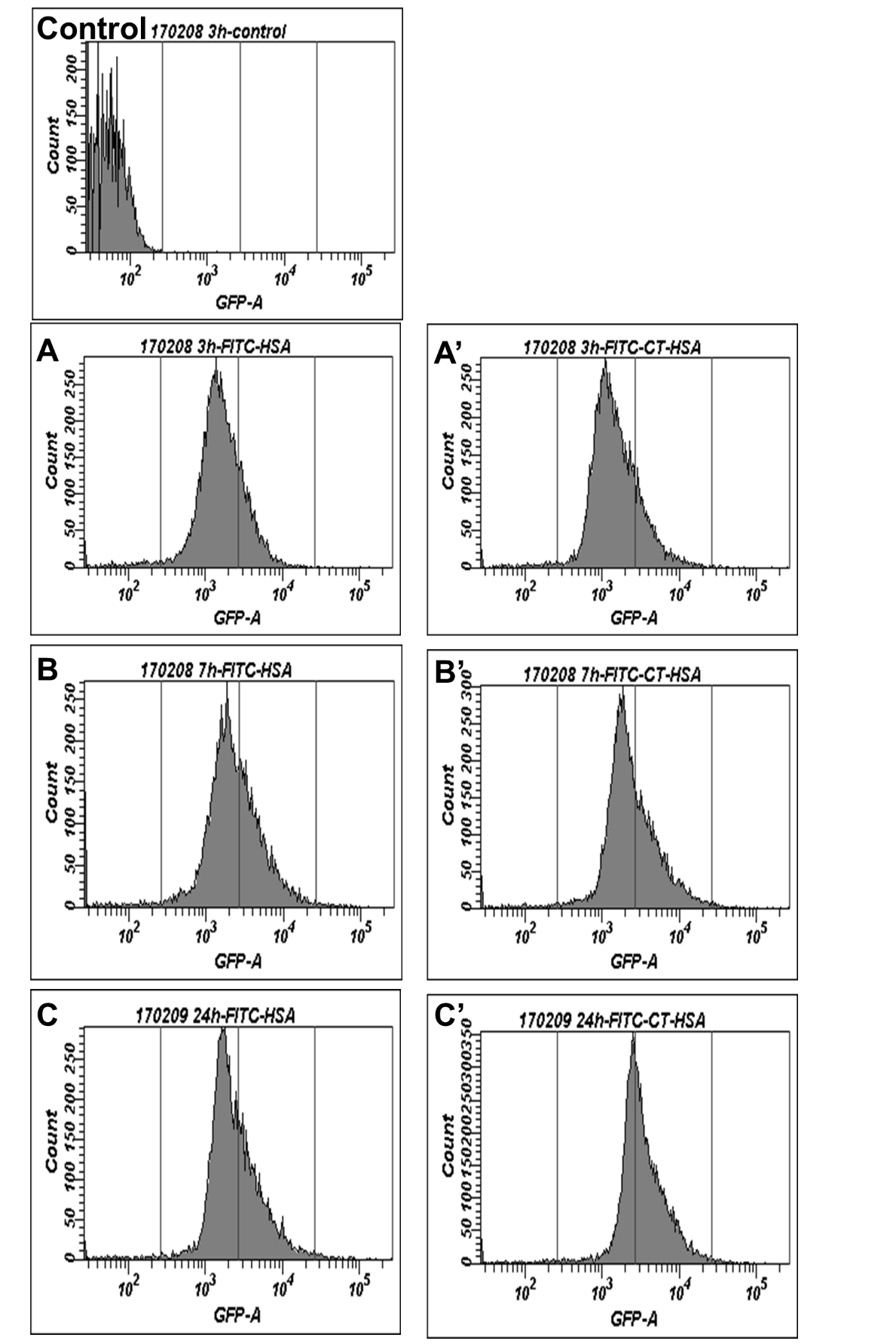


**Figure S6.** Time dependent of intracellular incorporation of FITC-labelled HSA to HeLa cells (A; 3h, B; 7h, C; 24h) or FITC-labelled CT-HSA to HeLa cells (A’; 3h, B’; 7h, C’; 24h). The data observed with flow cytometric analysis. Control means original fluorescence of HeLa cells. HeLa cells were seeded into a 35 mm dish at 1.0 × 10^4^ cells/cm^2^, and the concentration of FITC-labelled HSA and FITC-labelled CT-HSA was 10 μM.


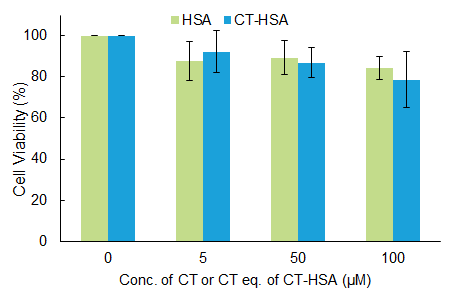


**Figure S7.** Cytotoxic effect of CT and CT-HSA on HeLa cells. HeLa cells were treated with CT or CT-HSA (CT equivalent 0~100 μM) Differences between CT and CT-HSA were analyzed by T. TEST (mean ± SD, n=5); *p< 0.01.
